# Population immunity to hepatitis B virus and infection marker seroprevalence in Belgrade, Serbia

**DOI:** 10.3389/fpubh.2026.1819814

**Published:** 2026-06-17

**Authors:** Anna Yu. Popova, Alesia Yu. Olkhovskaya, Luka Dragačević, Olga A. Petrova, Yulia V. Ostankova, Svetlana A. Egorova, Alexandr N. Schemelev, Marija Petrušić, Ekaterina V. Anufrieva, Anastasiya R. Ivanova, Irina V. Drozd, Ojuna B. Zhimbaeva, Darija K. Tepavcevic, Jelena Protić, Ekaterina M. Danilova, Angelica M. Milichkina, Valeri A. Ivanov, Oleg V. Kotsar, Edward S. Ramsay, Vyacheslav Y. Smolensky, Areg A. Totolian

**Affiliations:** 1 Federal Service for the Oversight of Consumer Protection and Welfare, Moscow, Russia; 2 Saint Petersburg Pasteur Institute, Saint Petersburg, Russia; 3 Institute of Virology, Vaccines, and Sera “Torlak”, Belgrade, Serbia

**Keywords:** antibodies, cohort study, hepatitis B, hepatitis B virus (HBV), herd immunity, Serbia, seroprevalence, vaccine-preventable diseases

## Abstract

Despite global progress in hepatitis B (HB) control, assessing herd immunity and the hidden burden of infection remains critical.

**Aim:**

This study aimed to comprehensively evaluate immunity to hepatitis B virus and HB marker prevalence in the Belgradian population (Serbia) based on infection/vaccination status and socio-demographic characteristics.

**Materials and methods:**

A cross-sectional study was conducted in Belgrade in May 2024, involving 2,533 individuals. Serological markers (HBsAg, anti-HBc, anti-HBs) were evaluated using ELISA. Statistical analysis included the calculation of 95% confidence intervals, the χ^2^ test, and Spearman's correlation analysis.

**Results:**

The overall HBsAg prevalence was 1.0% (25/2,533), classifying Serbia as a low-endemicity region. The seroprevalence of anti-HBc and anti-HBs was 8.7% (220/2,533) and 21.6% (547/2,533), respectively. A clear age-dependent trend was observed. Markers of infection (HBsAg, anti-HBc) increased significantly with age (*rs* = 0.823 and *rs* = 0.898, respectively) among adults, peaking in the 70+ years group for anti-HBc (22.2%, 42/189). In contrast, protective anti-HBs antibodies showed a negative correlation with age (*rs* = −0.75), being most prevalent in children and young adults (76.9%, 10/13, in the group 1–5 years). Vaccination coverage also showed a strong negative correlation with age (*rs* = −0.95), declining from 82.3% (93/113) among individuals under 18 to 4.6% (16/345) among retirees. These correlations clearly illustrate differing epidemiological profiles. They range from high natural viral circulation among older, unvaccinated cohorts to effective infection control through vaccine-induced immunity in the younger generation. A low level of population awareness was identified: only 1.1% (28/2,533) of respondents reported a history of HBV, whereas serological data indicated that 8.7% (220/2,533) of the population had markers of viral exposure.

**Conclusion:**

The study confirms the success of the universal child vaccination program in Serbia, which has led to the formation of a protected younger generation. However, a significant susceptible stratum persists among the adult population, alongside a high rate of undiagnosed infections, particularly in the older adults. These findings underscore the necessity of implementing targeted adult screening programs and optimizing vaccination strategies to achieve HB elimination.

## 1 Introduction

Viral hepatitis B (HB) infection remains a global public health problem, posing a significant threat to the world's population. According to the World Health Organization (WHO), approximately 2 billion people worldwide are infected with the hepatitis B virus (HBV), of whom over 250 million are living with chronic infection, which is a leading cause of liver cirrhosis and hepatocellular carcinoma (1). Despite impressive progress in prevention achieved through the introduction of mass vaccination, the disease burden remains high, particularly in a number of geographical regions (2–4) and among vulnerable population groups (5–7), including certain occupational categories (8, 9).

Serological markers of HBV infection, such as the surface antigen (HBsAg), antibodies (Abs) to the core antigen (anti-HBc), and Abs to the surface antigen (anti-HBs), provide comprehensive information about an individual's infection and vaccination status (10, 11). Comprehensive analysis of the prevalence and combination of these markers in a population makes it possible not only to assess the current level of viral circulation, but also to retrospectively analyze the history of the epidemic process, the effectiveness of vaccination programs, and the level of herd immunity (10, 11). The importance of such an analysis is underscored by the fact that the epidemiological situation is not static. Marker prevalence can be influenced by various socio-demographic and occupational factors. Age is one of the most significant predictors of infection risk and disease outcome. Certain professional activities are also associated with an increased risk of infection. These include healthcare workers, social service workers, and individuals in contact with blood or bodily fluids. Therefore, stratifying data by age, occupation, and vaccination status is essential for obtaining an accurate and representative epidemiological picture.

Before the introduction of universal hepatitis B vaccination in 2005, Serbia experienced an intermediate level of HBV infection with an HBsAg prevalence of about 2%−7% in the general population and mixed modes of transmission among infants, young children, and adults (12). As a member state of the WHO European Region, Serbia has made significant efforts to control the spread of HBV over the past decades. Mandatory immunization against HBV for newborn infants born to HBsAg-positive mothers started in 1988 (13–15). Universal at-birth hepatitis B vaccination was implemented in 2005, creating distinct epidemiological cohorts characterized by differing levels and mechanisms of immunity formation. Concerning the vaccination of healthcare workers, and others at risk born before 2005, the vaccination is still optional. Consequently, individuals born after 2005 pre-dominantly represent cohorts with systematic vaccine-induced immunity. Older age groups represent a mixture of unvaccinated individuals, selectively vaccinated high-risk groups, and those with naturally acquired immunity. A key element of the national strategy has been mandatory immunizations defined in the national immunization schedule introduced in 2005: newborns within 24 h of birth; a second dose at the age of 1 month; and a third dose at the age of 6 months. At the same time, a nationwide catch-up vaccination campaign was also implemented, targeting all school children aged 12 years.

This has led to a significant decrease in the incidence of acute infection among the younger generation (16). Over the 15-year period from 2003 to 2017, the crude incidence of acute HBV infection in Serbia decreased from 4.92 to 0.25 per 100 K population (17).

However, a significant proportion of the adult population in Serbia, particularly older age groups, was not covered by vaccination and could have been susceptible to infection in the pre-vaccination period.

General epidemiological data on hepatitis B in Serbia are available from national and WHO sources (15, 16). However, there is still a need for a detailed analysis of seroprevalence at the national level with a comprehensive assessment of the interplay between key markers of infection. Most studies focus on specific risk groups or limited regions, whereas large-scale population-based studies encompassing all age groups and various sectors of activity remain limited.

Obtaining contemporary population-based seroprevalence data is crucial for verifying the effectiveness of national immunization programs, identifying gaps in collective immunity, and planning targeted preventive measures. Despite the impressive progress achieved through universal childhood vaccination, the path toward the elimination of hepatitis B by 2030, as set by the World Health Organization (WHO), presents new challenges. Key challenges include identifying and vaccinating susceptible adults and detecting chronic infections in the adult population. In Serbia, where universal newborn vaccination was introduced in 2005, this has created a unique epidemiological dichotomy between the protected younger generation and the unvaccinated adult population. However, to develop targeted and cost-effective strategies for adult screening and vaccination, contemporary, comprehensive data on HBV seroprevalence with detailed stratification by age and vaccination status are essential. Existing studies in Serbia often focus on specific regions or risk groups, whereas large-scale, population-based studies encompassing the entire age spectrum and allowing for the assessment of herd immunity in a transitional epidemiological setting remain scarce. The administrative area of the City of Belgrade (the Belgrade District) covers 3,227 km^2^, accounting for 3.6% of Serbia's territory. According to the 2022 census, the population of the Belgrade District is 1.68 million, representing 22.5% of the national population, with 1.19 million inhabitants living in the urban core of the city itself. This makes Belgrade a key demographic and epidemiological sentinel for the entire country. The aim of this study was to conduct a comprehensive assessment of HBV immunity in the Belgradian population (Serbia) by analysis of serological marker prevalence and profile patterns.

## 2 Materials and methods

### 2.1 Study design and ethics approval

A cross-sectional randomized study, “Herd immunity to vaccine-preventable and other relevant infections in the Belgradian population”, was conducted in May, 2024 under a joint program between Rospotrebnadzor (Russia) and the Serbian Ministry of Health. It was approved by the relevant local ethics committees at the Saint Petersburg Pasteur Institute (St. Petersburg, Russia) and the Institute of Virology, Vaccines, and Sera “Torlak” (Belgrade, Serbia).

### 2.2 Participant recruitment and randomization

A broad public information campaign was conducted in national and regional media (including television, news websites, and social media) to promote the study. The campaign, presented as part of a scientific research project, invited interested persons from the general population of Belgrade to undergo a free laboratory screening. Media publications contained a direct link to the dedicated study web application (Saint Petersburg Pasteur Institute, St. Petersburg, Russia). The web application served as the central platform for initial screening and randomization. Upon accessing it, potential volunteers were presented with a detailed digital information sheet outlining the study's purpose, procedures, risks, benefits, data handling, and privacy policies. Those who agreed to proceed provided preliminary electronic consent and completed a core demographic questionnaire (collecting age, gender, and region of residence). Upon questionnaire completion, the application performed real-time selection using a built-in pseudorandom number generator seeded by the system clock. Each volunteer was assigned a random integer; only those for whom this integer was divisible by three proceeded further (inclusion probability *p* = 1/3). All other volunteers were excluded from the study. Selected volunteers were then assigned to one of nine pre-defined age strata based on their reported age. Each stratum was filled sequentially until its quota was reached, ensuring proportional age representation (see Sample Size Calculation and Stratification for census-based quota derivation). Once the quota for a given age stratum was met, the application stopped inviting further volunteers from that age group. This procedure constitutes a randomized quota sampling design: randomness is applied only to the initial screening step, while enrolment follows deterministic quota-based rules to ensure age representativeness. Only individuals selected by this algorithm were contacted and invited to a designated blood collection point for final enrollment. All other applicants were excluded from further stages of the study.

### 2.3 Inclusion and exclusion criteria

Participation in the study was entirely voluntary. The primary inclusion criterion was the provision of valid written informed consent at the blood collection point. Key exclusion criteria were: (1) the inability or unwillingness to provide such consent; (2) the presence of an acute illness at the time of the visit; or (3) current receipt of immunosuppressive therapy. These medical exclusions were implemented to minimize confounding effects on serological markers. All individuals meeting the requirements (selected via the web application, providing final written consent at the collection point, meeting the inclusion criteria, completing study procedures) were enrolled.

### 2.4 Informed consent process

Informed consent was obtained in a two-stage procedure. First, potential participants reviewed the digital information and provided preliminary electronic consent within the web application, which was required to complete the initial demographic questionnaire and be considered for random selection. Second, final written informed consent was obtained in person from selected volunteers by study staff at the blood collection point before any further procedures, such as completion of the detailed study questionnaire or blood sample collection.

### 2.5 Data collection

At the blood collection point, the following data were collected from all enrolled participants via a structured questionnaire administered at the center. In addition to age and gender, participants reported their field of activity, which was later categorized into broad groups (e.g., healthcare worker, education, office worker, student, retired, unemployed). Self-reported medical history included specific questions regarding a history of clinically diagnosed hepatitis B infection (yes/no/unsure) and hepatitis B vaccination status (vaccinated/not vaccinated/unsure). Furthermore, a history of potential parenteral exposures was assessed by asking participants about any prior surgical procedures and/or blood transfusions (both as yes/no). Data on vaccination status and prior hepatitis B infection were based on self-reporting by participants, supported by physical vaccination certificates when available. It is important to note that this method may be subject to recall bias, and for the majority of adult participants, it was not feasible to verify this information against formal medical records. These variables were selected for their established or potential epidemiological relevance to HB serostatus and were included in the subsequent stratified analyses, as presented in the Results section. The collection of anamnestic data and blood samples lasted for two full weeks in May, 2024.

### 2.6 Study population and sampling

#### 2.6.1 Sample size calculation and stratification

The required cohort size was calculated using standard epidemiological methodology for population-based seroprevalence studies, as previously described (18–21). In addition, the sampling framework was based on official census data for the population of Belgrade published by the Statistical Office of the Republic of Serbia (2011 Census, with updated population estimates) (22). These data were used to ensure adequate representativeness across pre-defined age groups. The pre-defined age stratification was intentionally designed to reflect standard demographic and epidemiological age categories commonly used in national surveillance and international seroprevalence studies. In the interpretation phase, these age groups were explicitly analyzed in the context of the historical implementation timeline of hepatitis B vaccination in Serbia (targeted vaccination since 1988, universal newborn vaccination since 2005).

#### 2.6.2 Cohort size and demographics

A total of 2,533 individuals were enrolled and stratified into nine age groups: 1–5 years (*n* = 13), 6–11 years (*n* = 43), 12–17 years (*n* = 62), 18–29 years (*n* = 249), 30–39 years (*n* = 501), 40–49 years (*n* = 688), 50–59 years (*n* = 468), 60–69 years (*n* = 320), and 70+ years (*n* = 189). As follows from the data, the cohort was dominated by middle-aged volunteers in the range of 30 to 59 years old, whose total share was 65.4% (95% CI: 63.5–67.2). In terms of gender, the cohort included 67.7% females and 32.3% males; the number of females in the cohort was 2-fold higher than males (23).

#### 2.6.3 Consideration of potential sampling bias

Although the cohort included a higher proportion of female participants (67.7%), this imbalance most likely reflects voluntary participation patterns and healthcare-seeking behavior, which has been consistently reported in population-based seroepidemiological studies. Importantly, hepatitis B serological markers are not considered to be strongly sex-dependent. Therefore, this imbalance is unlikely to have significantly biased the main outcomes of the study.

The study material consisted of plasma samples derived from whole blood collected using the anticoagulant EDTA. Examination of volunteers for hepatitis B serological markers involved the qualitative determination of HBsAg and total anti-HBc Abs in plasma using the commercial kits “DS-ELISA-HBsAg” and “DS-ELISA-ANTI-HBc” (NPO “Diagnostic Systems,” Russia), respectively, according to manufacturer instructions. To assess the strength of immunity to HBV, the prevalence and level of total anti-HBs Abs were evaluated quantitatively using the “DS-ELISA-ANTI-HBsAg” kit (NPO “Diagnostic Systems,” Russia), in accordance with the manufacturer's protocol.

Statistical data processing was carried out using GraphPad Prism 5.0 (GraphPad Software, Inc.) software package. Key statistical results were independently verified using R software (version 4.3.2). Microsoft Excel (Microsoft Corp.) was used for data organization, table preparation, and graphical presentation. To assess statistical error, the exact Cloepper-Pearson interval was applied. Results are presented with a 95% confidence interval (CI). For assessing the significance of differences in quantitative data during paired comparisons, either Fisher's exact test, or the Chi-square test with Yates' correction, was used depending on sample characteristics. A probability value of *p* < 0.05 was set as the threshold for statistical significance. Correlation analysis was conducted, taking into account compliance with parametric distribution, with calculation of Spearman rank correlation coefficients (*rs*). Differences were considered statistically significant when *p* < 0.05.

## 3 Results

**Figure 1:**
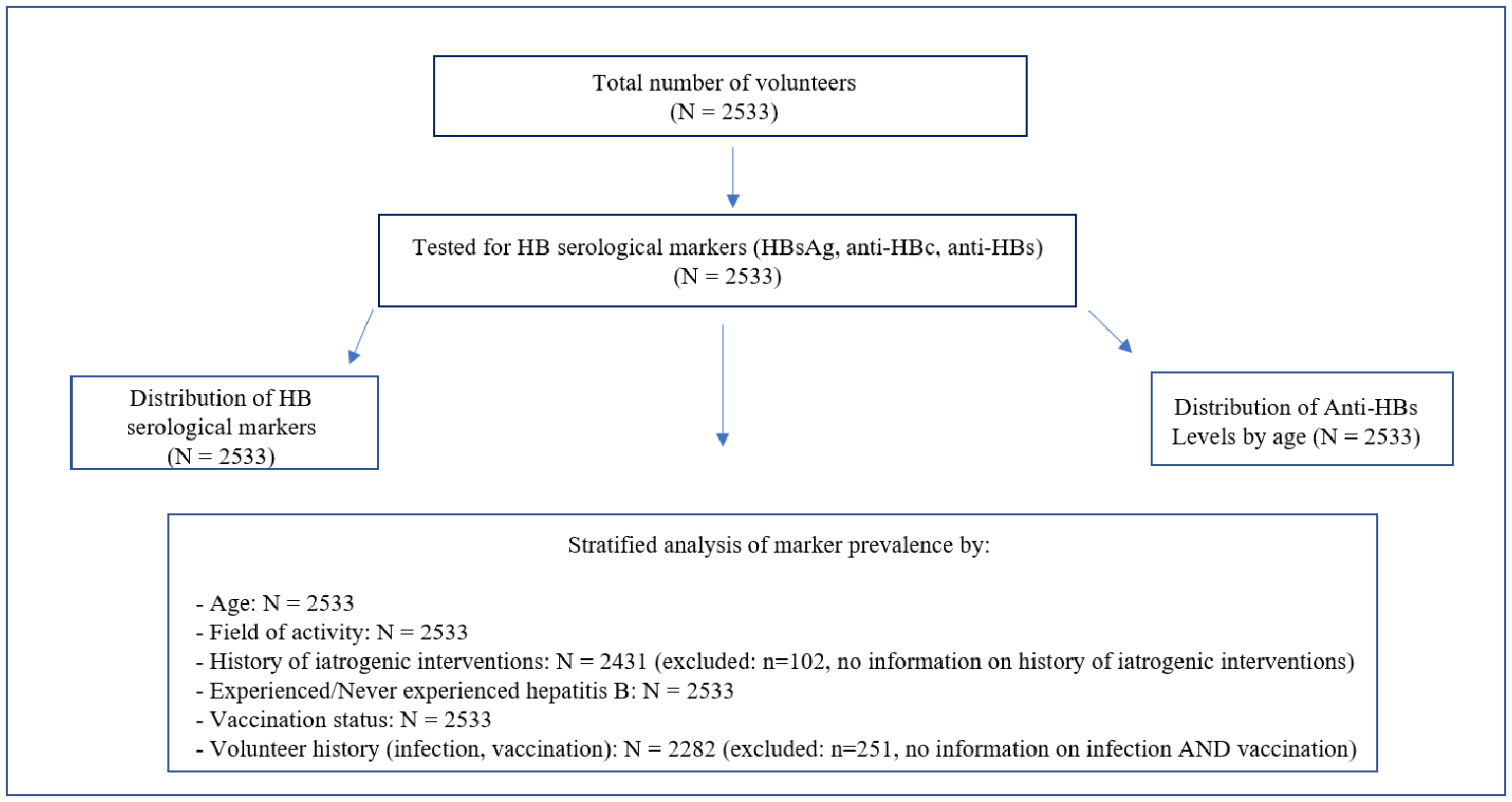
Flowchart of viral marker analyses and analytical subgroups. The demographic and clinical characteristics of the cohort are presented here to provide context for subsequent analyses.

The study population consisted of 2,533 volunteers from Belgrade. The selection of participant samples for each type of analysis (workflow) is presented visually in Figure 1.

The prevalence of HB serological markers shows a distinct age dependence. With increasing age, a sequential rise in the detection frequency of infection markers (HBsAg, anti-HBc) is observed, peaking in the older adult age groups. In contrast, the prevalence of anti-HBs Abs decreases with age, with the maximum level found in the group 1–5 years and the minimum in the group 60–69 years. HBsAg was detected in 25 individuals (1.0%, 95% CI: 0.7–1.5%), anti-HBs IgG Abs in 547 volunteers (21.6%, 95% CI: 20.0%−23.2%), and anti-HBcore in 220 volunteers (8.7%, 95% CI: 7.7–9.8%). The prevalence of serological markers across different age groups is presented in Table 1.

**Table 1:** Serological marker prevalence by volunteer age group.

Age group, years	*N*	HBsAg			Anti-HBc			Anti-HBs		
		*n*	%	95% CI	*n*	%	95% CI	*n*	%	95% CI
1–17	118	0	0.0	0.0–3.1	10	8.5	4.7–14.9	42	35.6^*^	27.5–44.6
1–5	13	0	0.0	0.0–24.7	1	7.7	1.4–33.3	10	76.9^*^	49.7–91.8
6–11	43	0	0.0	0.0–8.2	5	11.6	5.1–24.5	17	39.5^*^	26.4–54.4
12–17	62	0	0.0	0.0–5.8	4	6.5	2.5–15.4	15	24.2	15.2–36.2
18–29	249	1	0.4	0.1–2.2	13	5.2	3.1–8.7	145	58.2^*^	52.0–64.2
30–39	501	2	0.4	0.1–1.4	27	5.4	3.7–7.7	102	20.4	17.1–24.1
40–49	688	5	0.7	0.3–1.7	43	6.3	4.7–8.3	103	15.0^**#**^	12.5–17.8
50–59	468	7	1.5	0.7–3.1	45	9.6	7.3–12.6	76	16.2^**#**^	13.2–19.9
60–69	320	8	2.5	1.3–4.9	40	12.5	9.3–16.6	42	13.1^**#**^	9.9–17.3
70+	189	2	1.1	0.3–3.8	42	22.2^*^	16.9–28.7	37	19.6	14.5–25.8
Total	2,533	25	1.0	0.7–1.5	220	8.7	7.7–9.8	547	21.6	20.0–23.2

^*^Significantly higher than the total value. ^**#**^Significantly lower than the total value; *p* < 0.05 for all comparisons.

Analysis of HBsAg prevalence revealed a clear age-related dynamic. No cases of HBsAg positivity were detected among children or adolescents aged 1–17 years (0%). Among the adult population, a trend of increasing HBsAg detection frequency with age was observed, ranging from 0.4% (95% CI: 0.1–2.2) in the group 18–29 years to 2.5% (95% CI: 1.3–4.9) in the group 60–69 years. No significant differences in HBsAg frequency were found between adult age groups (*p* > 0.05). It should be noted, however, that the sample size in the youngest pediatric subgroups, particularly ages 1–5 years (*n* = 13), is very limited, which affects the precision of prevalence estimates in these cohorts.

Seroprevalence of anti-HBc increased with age, from 8.5% (95% CI: 4.7–14.9) in minors (1–17 years) to 22.2% (95% CI: 16.9–28.7) in individuals aged 70+ years (Table 1). Among adults, the lowest prevalence was observed in the group 18–29 years (5.2%; 95% CI: 3.1–8.7). No significant differences were found between adjacent age groups under 50 years old. A significant increase was noted starting from the group 50–59 years, whose prevalence (9.6%) was higher than in the group 40–49 years (6.3%) (χ^2^ = 4.02, *p* = 0.045). The combined prevalence in the age bracket 50–69 years (10.8%) was significantly higher than in the bracket 18–49 years (5.8%) (χ^2^ = 17.63, *p* < 0.0001) and significantly lower than in the group 70+ years (χ^2^ = 16.63, *p* < 0.0001).

Anti-HBs seroprevalence featured a complex, non-linear dependence on age, from 76.9% (95% CI: 49.7–91.8) in children aged 1–5 years to 13.1% (95% CI: 9.9–17.3) in the group 60–69 years (Table 1). In the pediatric cohort (1–17 years), prevalence decreased significantly from 76.9% to 39.5% between the groups 1–5 and 6–11 years (Fisher's exact test, *p* = 0.027). Interpretation of this trend in young children requires caution due to the very limited sample size (*n* = 13 in the 1–5 years group). Among adults, anti-HBs prevalence peaked at 58.2% in the group 18–29 years, followed by a sharp decline to 20.4% in the group 30–39 years (χ^2^ = 106.31, *p* < 0.0001). No significant differences were observed between adult age groups aged 30 years and older.

**Figure 2:**
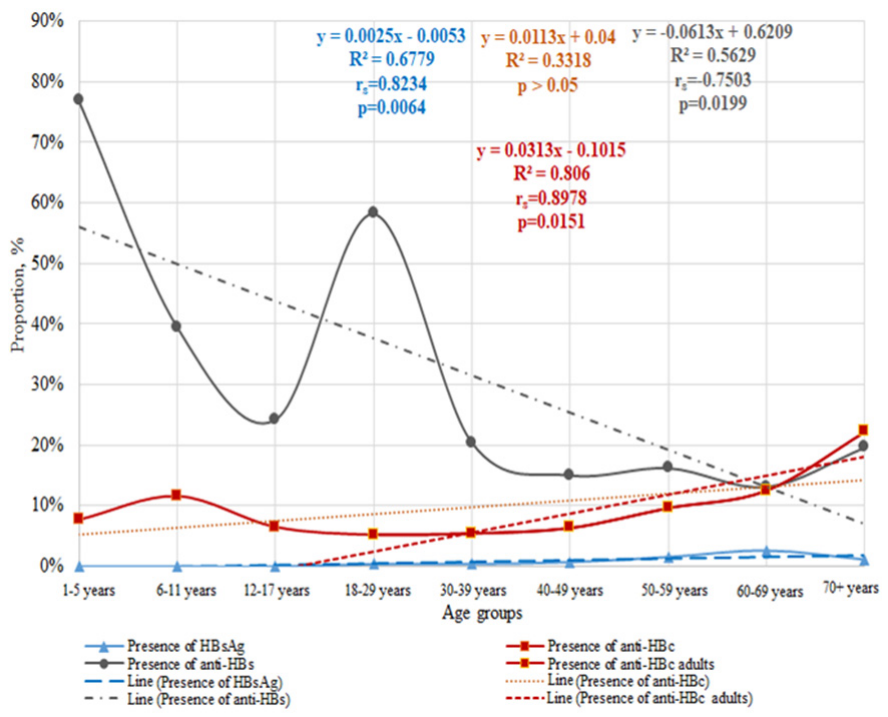
Correlation of marker frequency with volunteer age.

Spearman rank correlation showed significant associations between age and HBV marker detection frequency (Figure 2). A strong positive correlation was observed between age and HBsAg prevalence (*rs* = 0.823, *p* = 0.006), indicating a linear increase in the frequency of viral carriage with age. For anti-HBc, a strong positive correlation with age was found in the analysis including only adults (*rs* = 0.898, *p* = 0.015). However, this correlation ceased to be statistically significant when the pediatric population was included in the analysis. For anti-HBs, a strong negative correlation with age was identified (*rs* = −0.75, *p* = 0.020), reflecting a decrease in immune coverage with increasing age.

Serological profiles (combinations of HBsAg, anti-HBc, and anti-HBs) provide crucial information for distinguishing between vaccine-induced and infection-acquired immunity, as well as for characterizing the stage of HBV infection, insofar as anti-HBs Abs alone serve as a marker of the immune response following vaccination or past infection. Therefore, in our cohort, the contribution of these two sources to overall anti-HBs seropositivity was assessed by analyzing combinatorial profiles with anti-HBc. The distribution of all profiles in the cohort is presented in Table 2.

**Table 2:** Distribution of serological markers in the study group.

Hepatitis B markers (combinatorial serological profile)	Prevalence (*N* = 2,533)		
	*n*	%	95% CI
HBsAg only	11	0.4	0.2–0.8
Anti-HBs only	435	17.2	15.8–18.7
Anti-HBc only	100	3.9	3.3–4.8
HBsAg, anti-HBs	2	0.1	0.0–0.3
HBsAg, anti-HBc	10	0.4	0.2–0.7
HBsAg, anti-HBs, anti-HBc	2	0.1	0.0–0.3
Anti-HBs, anti-HBc	108	4.3	3.5–5.1
Any of the following: HBsAg, anti-HBs, anti-HBc	668	26.4	24.7–28.1
The aforementioned markers are absent	1,865	73.6	71.9–75.3

**Figure 3:**
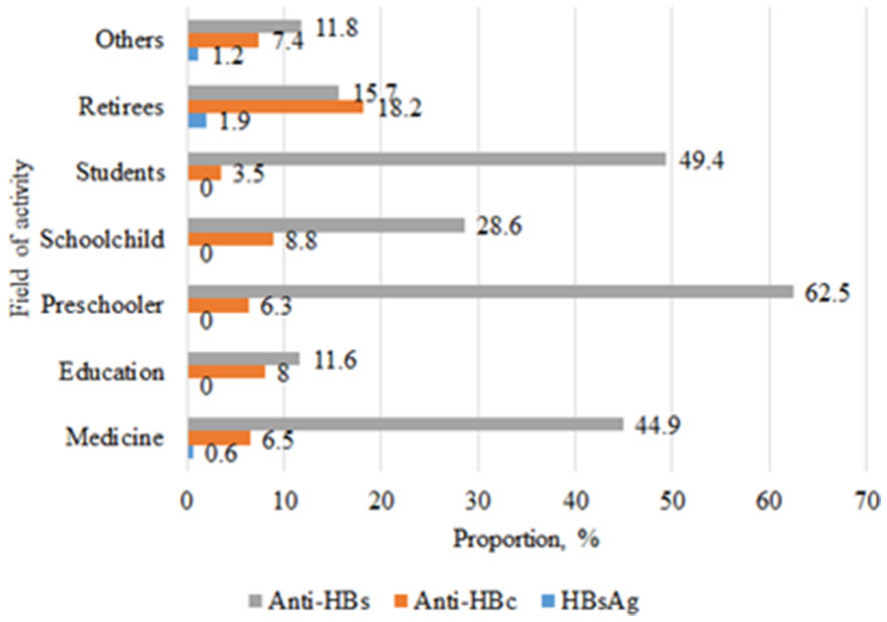
Hepatitis B markers in epidemiologically significant groups.

Despite the overall low endemicity of hepatitis B in Serbia, the risk of infection varies significantly by activity. Professional activities associated with a higher risk of contact with potential sources of infection are a concern. Analysis of serological marker prevalence among different occupational groups is crucial for assessing the effectiveness of existing prevention programs and identifying populations requiring additional protective measures. The results of assessment of HBsAg, anti-HBc, and anti-HBs detection frequency among volunteers from different fields of activity (healthcare, education, business, retirees, students, pre-schoolers, schoolchildren, unemployed, and others) are presented below (Figure 3). The full breakdown is available in Supplementary Table S1.

Marker prevalence varied across activity groups (Figure 3). The highest frequencies of HBsAg and anti-HBc were observed among retirees, while the highest frequency of anti-HBs was found in pre-schoolers, schoolchildren, students, and healthcare workers. The estimates for pre-schoolers (*n* = 16) and schoolchildren (*n* = 91), while indicative, are based on relatively small sample sizes and should be interpreted with this limitation in mind.

We assessed the association between “a history of surgical interventions or blood transfusions” and the prevalence of HBV serological markers. Only those volunteers who were certain about the presence or absence of medical interventions were considered. No significant differences were found between individuals with or without such a history (Supplementary Table S2). Self-reported HBV infectious history was compared with serological results (Table 3; detailed age breakdown is shown in Supplementary Table S3).

**Table 3:** Marker prevalence by volunteer self-report history.

Infectious status	*N*	HbsAg			Anti-HBc			Anti-HBs		
		*n*	%	95% CI	*n*	%	95% CI	*n*	%	95% CI
Experienced hepatitis B	28	6	21.4^*^	10.2–39.5	22	78.6^*^	60.5–89.8	12	42.9^*^	26.5–60.9
Never experienced hepatitis B	2,468	17	0.7	0.4–1.1	189	7.7	6.7–8.8	527	21.4	19.8–23.0
Information not available	37	2	5.4	1.5–17.7	9	24.3^*^	13.4–40.1	8	21.6	11.4–37.2
Total	2,533	25	1.0	0.7–1.5	220	8.7	7.6–9.9	547	21.6	20.0–23.25

^*^Significantly higher than the total value. ^**#**^Significantly lower than the total value; *p* < 0.05 for all comparisons.

Analysis of the self-reported infectious status of respondents showed that only 1.1% of participants reported having had HB. The proportion of such responses increased predictably with age: from 0% (those < 30 years) to 5.8% (those ≥70 years). The vast majority of respondents (97.4%) considered themselves as not having had HB, while 1.5% did not have this information available. It is important to note that among children and adolescents (1–17 years), not a single case of reported past infection was recorded.

Comparison of the infectious status self-reports with objective serological data revealed a critically low level of awareness among respondents about past infection. Among the 28 individuals who reported having had HB, markers of current infection (HBsAg) were detected in 21.4%, and anti-HBc in 78.6%, confirming the accuracy of their responses. However, among the 2,468 respondents who considered themselves as not having had the disease, HBsAg was detected in 0.7%, and anti-HBc in 7.7%, indicating asymptomatic infection and a lack of diagnosis. In the group with unknown infectious status (37 individuals), anti-HBc was detected in 24.3%, and HBsAg in 5.4%. It is important to note that the overall seroprevalence of anti-HBc in the sample (8.7%) was 8.9-fold higher than the proportion of individuals who were aware of their past infection (1.1%). Table 4 presents data on volunteer vaccination status across different age groups. Only those volunteers who were certain about the presence or absence of HB vaccination were considered.

**Table 4:** Hepatitis B vaccination status by volunteer age group.

Age group, years	*N*	Vaccinated			Not vaccinated		
		*n*	%	95% CI	*n*	%	95% CI
1–17	113	93	82.3^*^	74.2–88.2	20	17.7^**#**^	11.8–25.8
1–5	11	9	81.8^*^	52.3–94.9	2	18.2^**#**^	5.1–47.7
6–11	40	30	75.0^*^	59.8–85.8	10	25.0^**#**^	14.2–40.2
12–17	62	54	87.1^*^	76.6–93.3	8	12.9^**#**^	6.7–23.4
18–29	222	147	66.2^*^	59.8–72.1	75	33.8^**#**^	27.9–40.2
30–39	440	159	36.1	31.8–40.7	281	63.9	59.3–68.2
40–49	626	169	27.0	23.7–30.6	457	73.0	69.4–76.3
50–59	431	108	25.1	21.2–29.4	323	74.9	70.6–78.8
60–69	299	26	8.7^**#**^	6.0–12.4	273	91.3^*^	87.6–94.0
70+	176	9	5.1^**#**^	2.7–9.4	167	94.9^*^	90.6–97.3
Total	2,307	711	30.8	29.0–32.7	1,596	69.2	67.3–71.0

^*^Significantly higher than the total value. ^**#**^Significantly lower than the total value; *p* < 0.05 for all comparisons.

**Figure 4:**
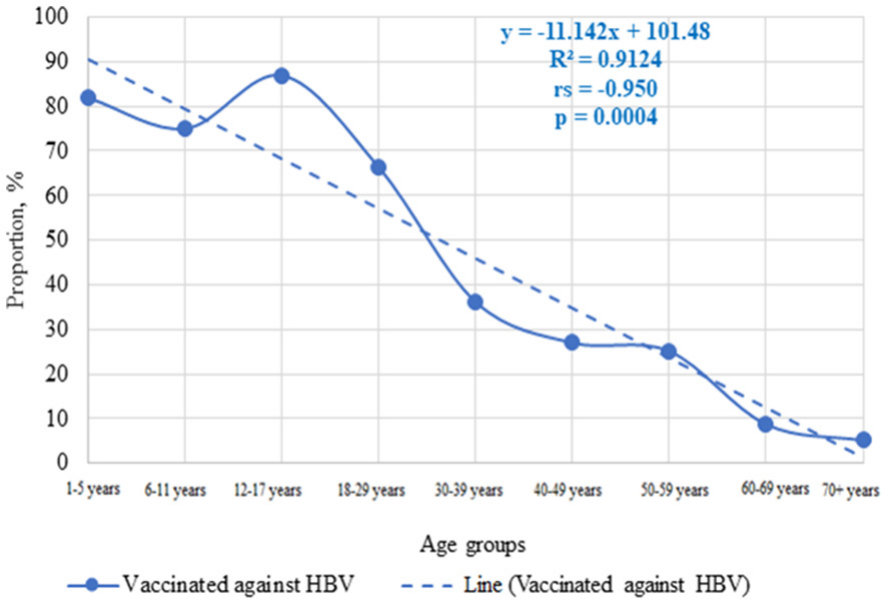
Correlation between proportion vaccinated and age group.

Vaccination coverage based on self-report showed a steep age-dependent decline from 82.3% (95% CI: 74.2–88.2) in children and adolescents (1–17 years) to 5.1% (95% CI: 2.7–9.4) in those aged ≥70 years (Table 4). Estimates for the youngest pediatric subgroups (e.g., 1–5 years, *n* = 13) should be interpreted with caution due to limited sample size. Statistical analysis revealed an exceptionally strong negative correlation between vaccination coverage and age group (*rs* = −0.95, *p* = 0.0004) (Figure 4). A linear regression model (y = −11.142 x + 101.48) confirmed that age is a powerful predictor of vaccination status, explaining 91.24% of the variation (R^2^ = 0.9124). This mathematically confirms the visual trend, namely a sharp decline in vaccination coverage with increasing age.

Analysis of vaccination status by field of activity revealed extremely heterogeneous coverage levels. The highest rates were observed in groups covered by routine immunization (preschoolers, schoolchildren, students) and among healthcare workers (73.3%). The lowest coverage was recorded among retirees (4.6%) and education workers (16.1%). The estimates for pre-schoolers (*n* = 12) and schoolchildren (*n* = 90) are based on limited sample sizes and require cautious interpretation. The full breakdown is available in Supplementary Table S4.

Table 5 presents the results of serological testing for HB markers among vaccinated and unvaccinated individuals.

**Table 5:** Hepatitis B viral markers by vaccination status.

Vaccination status	*N*	HBsAg			Anti-HBc			Anti-HBs		
		*n*	%	95% CI	*n*	%	95% CI	*n*	%	95% CI
Vaccinated against HBV	711	5	0.7	0.3–1.6	44	6.2	4.6–8.2	358	50.4^*^	46.7–54.0
Not vaccinated against HBV	1,596	18	1.1	0.7–1.8	164	10.3	8.9–11.9	150	9.4^**#**^	8.1–10.9
Information not available	226	2	0.9	0.1–3.2	12	5.3	2.8–9.1	39	17.3	12.6–22.8
Total	2,533	25	1.0	0.7–1.5	220	8.7	7.7–9.8	547	21.6	20.0–23.2

^*^Significantly higher than the total value. ^**#**^Significantly lower than the total value. *p* < 0.05 for all comparisons.

Serological analysis revealed substantial differences in HB marker profile between vaccinated and unvaccinated individuals. The prevalence of anti-HBs was markedly higher in the vaccinated group (50.4%) compared to the unvaccinated (9.4%) (χ^2^ = 478.05, *p* < 0.0001). Conversely, anti-HBc prevalence was higher in the unvaccinated group (10.3% vs. 6.2%; χ^2^ = 9.52, *p* = 0.002). HBsAg prevalence did not differ significantly between the groups.

For a combined analysis of vaccination and infection history, only participants who were certain about both their vaccination status and their history of HBV infection were included (*N* = 2,282). These individuals were categorized into four groups based on self-reported history of vaccination and infection: never infected but vaccinated (NIV); neither infected nor vaccinated (NINV, “naive”); experienced infection and vaccinated (IV); and experienced infection and not vaccinated (INV). The distribution of these categories by age group is shown in Supplementary Table S5.

The analysis revealed a pronounced age-related polarization (Supplementary Table S). In the group 1–17 years, the majority (82.3%) were vaccinated without evidence of infection (NIV), with no cases of past infection (INV/IV). Conversely, in adults over 60 years, most individuals were neither infected nor vaccinated (NINV: 89.4% in 60–69 years and 89.0% in 70+ years), while those vaccinated (NIV) constituted a small minority. Cases of past infection (INV) were found only in adults over 30 years, with a low prevalence (1.1% overall) that was somewhat higher in the oldest group (5.8% in 70+ years).

The prevalence of HBV markers within each immune status category is shown in Table 6. Among INV individuals (*n* = 26), HBsAg was detected in 23.1%, anti-HBc in 84.6%, and anti-HBs in 46.2%. Among NIV individuals (*n* = 708), anti-HBs was detected in 50.4%, while anti-HBc was found in only 6.1%. In the NINV group (*n* = 1,546), the prevalence of anti-HBs was 8.6%, and anti-HBc was 8.8%. No markers were detected in the IV group (*n* = 2). The detailed prevalence of serological markers within each immune status category (NIV, NINV, INV, IV) by age group is provided in Supplementary Tables 6–8.

**Table 6:** HBsAg, anti-HBc, anti-HBs detection frequency by volunteer history (infection, vaccination).

Markers	*N*	INV				IV				NINV				NIV			
		*N*	*n*	%	95% CI	*N*	*n*	%	95% CI	*N*	*n*	%	95% CI	*N*	*n*	%	95% CI
HBsAg	2,282	26	6	23.1	11.0–42.1	2	0	0.0	0.0–84.2	1,546	11	0.7	0.4–1.3	708	5	0.7	0.3–1.6
Anti-HBc	2,282	26	22	84.6	66.5–93.9	2	0	0.0	0.0–84.2	1,546	136	8.8	7.5–10.3	708	43	6.1	4.5–8.1
Anti-HBs	2,282	26	12	46.2	28.8–64.5	2	0	0.0	0.0–84.2	1,546	133	8.6	7.3–10.1	708	357	50.4	46.7–54.1

**Figure 5:**
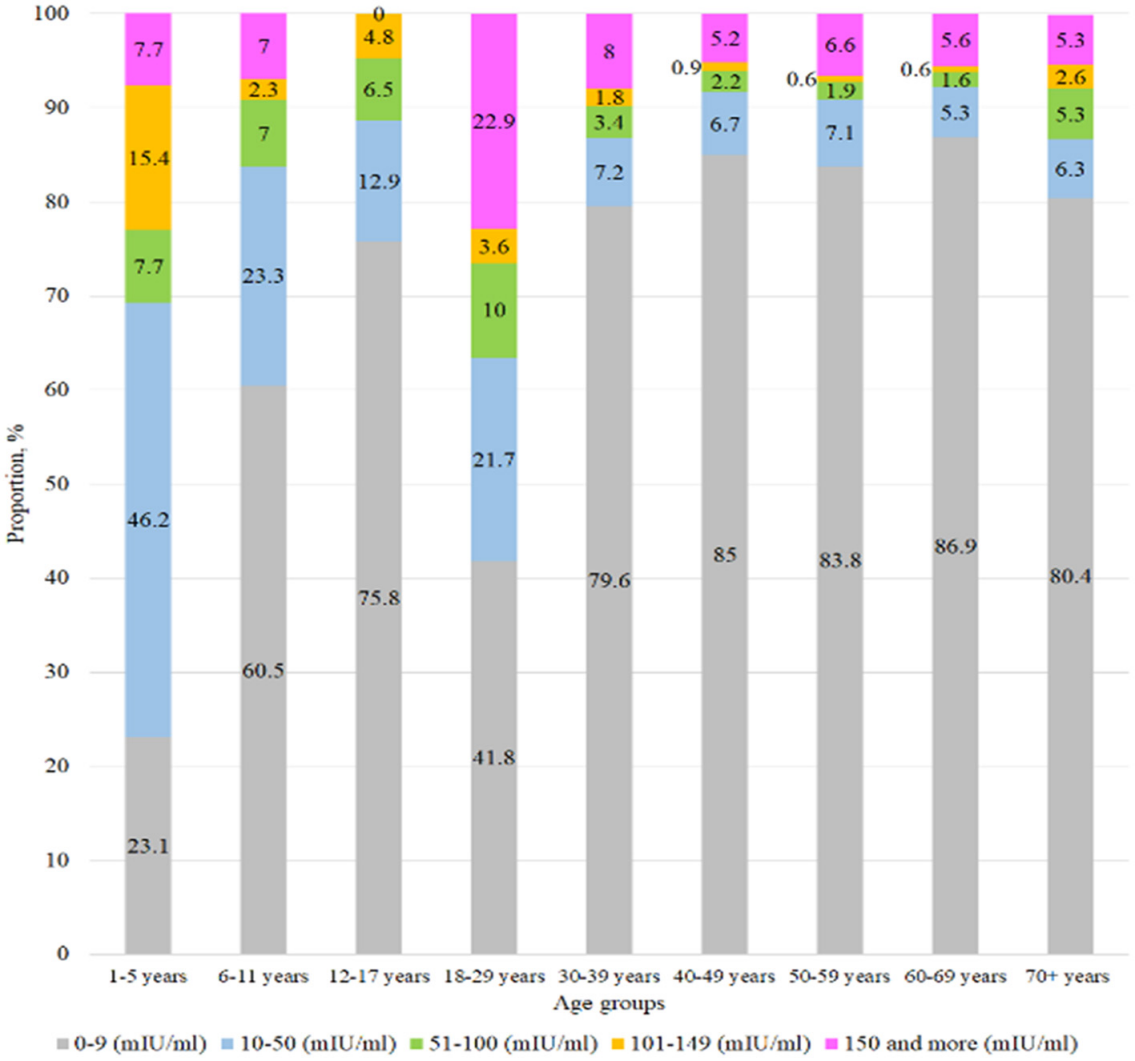
Anti-HBs levels by volunteer age group.

Quantitative anti-HBs antibody levels were analyzed across age groups (Figure 5; detailed data are shown in Supplementary Table S9). The proportion of individuals with undetectable anti-HBs levels (< 10 mIU/ml) increased with age from 23.1% in children aged 1–5 years to over 86% in groups aged 40 years and older. Conversely, high antibody levels (≥100 mIU/ml) were most prevalent in the groups 18–29 years (23.0%) and 1–5 years (23.1%). The share of individuals with any detectable protective titer (>10 mIU/ml) was highest in the groups 1–5 years (76.9%) and 18–29 years (58.2%).

Anti-HBs levels were also compared based on vaccination status (Table 7). As shown in Table 7, 50.4% of vaccinated individuals had detectable anti-HBs (>10 mIU/ml), compared to 9.4% of unvaccinated individuals. High anti-HBs levels (≥100 mIU/ml) were found in 18.0% of the vaccinated and 3.6% of the unvaccinated. The majority of unvaccinated individuals (90.6%) had antibody levels below 10 mIU/ml.

**Table 7:** Anti-HBs levels in volunteers by HBV vaccination status.

Vaccination status	*N*	0–9 (mIU/ml)			10–50 (mIU/ml)			51–100 (mIU/ml)			101–149 (mIU/ml)			≥ 150 (mIU/ml)		
		*n*	%	95% CI	*n*	%	95% CI	*n*	%	95% CI	*n*	%	95% CI	*n*	%	95% CI
Vaccinated against HBV	711	353	49.6^**#**^	46.0–53.3	150	21.1^*^	18.3–24.2	54	7.6^*^	5.9–9.8	26	3.7^*^	2.5–5.3	128	18.0^*^	15.4–21.0
Not vaccinated against HBV	1,596	1,446	90.6^*^	89.1–91.9	55	3.4	2.7–4.5^#^	27	1.7^**#**^	1.2–2.5	11	0.7	0.4–1.2	57	3.6^#^	2.8–4.6
Total	2,307	1,799	78.0	76.2–79.6	205	8.9	7.8–10.1	81	3.5	2.8–4.3	37	1.6	1.2–2.2	185	8.0	7.0–9.2

^*^Significantly higher than the total value. ^**#**^Significantly lower than the total value. *p* < 0.05 for all comparisons.

## 4 Discussion

This study provides a comprehensive population-based assessment of hepatitis B immunity and serological marker prevalence in Belgrade, Serbia. The obtained data make it possible not only to ascertain the current level of endemicity, but also to trace the historical dynamics of viral spread, assess the effectiveness of implemented preventive measures, and identify systemic gaps requiring immediate attention from the healthcare system. The results show a clear age-dependent pattern reflecting historical changes in vaccination policy and viral circulation.

The success of the universal childhood hepatitis B vaccination program is primarily evidenced by the absence of infection markers (HBsAg, anti-HBc) in younger age groups, rather than by anti-HBs seropositivity alone. However, a substantial susceptible stratum persists among adults, particularly in older age groups, underscoring the need for targeted screening and adult vaccination strategies.

### 4.1 Transformation of the epidemiological landscape: from historical circulation to a controlled situation

Serbia is a low-endemicity region for HB (HBsAg 1.0%, anti-HBc 8.7%). However, a profound generational gap exists, reflecting the impact of vaccination. This “epidemiological divide” is typical of successful universal immunization (24). The absence of HBsAg carriage in the group 1–17 years (0%) reflects the success of routine vaccination in interrupting viral transmission among younger generations. This aligns with global trends in countries with universal programs (25–29). This success contrasts with the identified strong positive correlation between age and HBsAg or anti-HBc prevalence (*rs* = 0.823 and *rs* = 0.898) observed among adults. The gradual increase in HBsAg and anti-HBc frequency serves as an “epidemiological archive”, reflecting the cumulative risk of infection faced by older generations in the pre-vaccination era when the level of viral transmission was substantially higher.

However, when younger groups are included in the analysis, the correlation does not hold insofar as these individuals have not been affected by historical viral circulation. The detection of anti-HBc in 8.5% of children/adolescents suggests some persistent viral circulation (30). However, these findings must be interpreted with great caution. Given the very small sample size of the pediatric subgroups, the calculated prevalence values feature considerable statistical uncertainty, as reflected in the wide confidence intervals. Therefore, any conclusions about persistent viral circulation in these age groups based solely on these data are preliminary and require confirmation in larger, population-based studies. Overall, the low and stable anti-HBc seroprevalence levels among young adults (18–39 years) and children confirm that the introduction of vaccination, alongside universal blood donor screening and adherence to infection control measures, has led to a sharp reduction in the intensity of HBV transmission over the last 20–30 years.

The population's serological profile, reflected as marker combinations, definitively confirms this transitional phase. The dominance of the “anti-HBs only” profile (17.2%), a marker primarily of post-vaccination immunity, and the extremely low prevalence of potentially infectious profiles (0.4% HBsAg+/anti-HBc+), indicate a shift from natural viral circulation to a controlled situation maintained by vaccine prophylaxis. The vast majority of the population (73.6%) remains immunologically naive. On one hand, this testifies to the success of control efforts. On the other, it identifies a substantial risk group for the future.

### 4.2 Immune coverage: successes, gaps, and the need for monitoring

The extremely high prevalence of protective anti-HBs in the group 1–5 years (76.9%) is a direct consequence of the high immunogenicity of routine infant immunization (28) and serves as a measure of its short-term effectiveness, with anti-HBs levels ≥10 IU/L considered seroprotective (31–35). The subsequent sharp decline among children (6–11 years, 39.5%) and adolescents (12–17 years, 24.2%) provides direct evidence of waning vaccine-induced humoral immunity in these younger age groups (28, 36, 37), raising legitimate questions about the durability of vaccine-induced protection. However, long-term follow-up studies indicate that despite waning antibody titers, functional immune memory persists, as evidenced by a robust anamnestic response to a booster dose in over 90% of individuals vaccinated in infancy (29). This phenomenon may be, to some extent, an artifact due to the small size of the subgroups.

The unexpectedly high share of individuals with anti-HBs in the group 18–29 years (58.2%) has a combined explanation. In addition to the persistent effect of childhood vaccination, contributions also come from other sources: booster campaigns among adolescents; vaccination among indicated occupations (healthcare workers, students); or prior travel to regions with high HB endemicity. This direct serological comparison between adolescents (24.2%) and young adults (58.2%) is essential. It highlights that initial vaccine-induced immunity declines, but can be recovered or enhanced through adolescent and young adult booster programs, underscoring different vaccination strategy needs across the lifespan. The sharp decline in the indicator in the group 30–39 years (20.4%), and its consistently low level among individuals over 40 years old, mark a boundary between generations (covered and not covered by routine childhood immunization).

The higher anti-HBs prevalence in those aged ≥70 years (19.6%) compared to preceding age groups is best explained by the serological profile of resolved infection: this cohort also has the highest anti-HBc prevalence (22.2%), indicating a high proportion of individuals with past successfully resolved infection (anti-HBc+/anti-HBs+ profile). This interpretation is supported by the significant negative correlation of anti-HBs with age (*rs* = −0.750), which aligns with the global trend of decreasing immune coverage in older age groups not covered by routine vaccination (26). This opposing trend (steep age-related decline in anti-HBs alongside an increase in anti-HBc and the “anti-HBc only” profile) is a hallmark of populations with high historical HBV exposure (38). Additionally, older individuals may have a lower immune response to vaccination due to immunosenescence (39, 40).

High coefficient of determination values (*R*^2^ = 0.678 for HBsAg and *R*^2^ = 0.806 for anti-HBc in adults) indicate that age is a key factor determining the prevalence of these markers. For anti-HBs (*R*^2^ = 0.563), other factors besides age, such as vaccination status and individual immune response characteristics also play a significant role. Nevertheless, considering the overlapping confidence intervals, the observed increase could also be a consequence of statistical variability, requiring further research to determine the precise causes of this trend.

### 4.3 Vaccination: achievements and shortcomings

Self-reported vaccination coverage declines steeply with age, mirroring the introduction of universal immunization. The sharp drop between age groups 18–29 and 30–39 years marks the generational divide, with an exceptionally strong negative correlation between age and vaccination status (*rs* = −0.95, *R*^2^ = 0.9124).

Among healthcare workers, despite high vaccination coverage, anti-HBs seropositivity (44.9%) remains suboptimal for a high-risk group (41, 42). Even more concerning is the low protection among education workers, whose immunity levels are comparable to the general population.

Nevertheless, vaccinated individuals have significantly higher anti-HBs and lower anti-HBc prevalence, confirming vaccine effectiveness in inducing protection and preventing infection (including asymptomatic cases). However, the detection of infection markers (HBsAg, anti-HBc) in some vaccinated individuals may indicate pre-vaccination infection, occult HBV (43, 44), or, less likely, immune-escape variants (45, 46). Co-detection of HBsAg and anti-HBs (0.16% of the cohort, 16% of HBsAg+ individuals) is a recognized phenomenon, often explained by vaccination of pre-existing chronic carriers (47–53), which highlights the limitation of vaccination in curing established infection and underscores the importance of pre-vaccination screening.

The anti-HBs seropositivity of only 50.4% among vaccinated individuals highlights the clinically significant issue of non-response or hypo-response, which is multifactorial (genetic pre-disposition, comorbidities, immunosenescence, suboptimal vaccine response) (54–60). For established non-responders, standard revaccination is often ineffective, necessitating personalized strategies (e.g., alternative schedules, adjuvants) (61–63). This challenge is compounded by the potential circulation of immune-escape variants (64). These findings provide a strong argument for implementing routine post-vaccination serological testing (34), particularly for healthcare workers and other high-risk groups, with protocols for alternative vaccination in non-responders. Without such a feedback loop, formal vaccination coverage cannot be considered a guarantee of individual or herd protection.

Anti-HBs in 9.4% of unvaccinated individuals likely stems from unrecorded vaccination or past resolved infection (59). Quantitative analysis showed a polarized titer distribution, with fundamental differences between vaccinated and unvaccinated groups. High titers in the latter may signal recent infection.

### 4.4 Occupational risks and iatrogenic safety

Seroprevalence across activity groups reflects both current vaccination efforts and historical exposure. The high anti-HBs levels among healthcare workers, students, and children highlight successful targeted and routine immunization. In contrast, the elevated anti-HBc frequency seen among retirees and military personnel indicates a historically high burden of infection in these cohorts. Notably, no significant differences in marker prevalence were found between individuals with or without a history of surgery or transfusion, indicating that modern infection control protocols have effectively mitigated iatrogenic HBV risk (65). Thus, under modern conditions, a history of medical interventions has ceased to be a significant risk factor for HBV infection, and the comparable anti-HBc prevalence rates likely reflect general population trends and risk factors unrelated to medical procedures. The obtained results are encouraging and testify to the high standard of safety of medical care provided in the country.

### 4.5 The hidden epidemic: a critical gap between subjective perception and reality

One of the most concerning findings is the profound gap between self-assessment and serological data. While 97.4% of respondents considered themselves never infected with hepatitis B, objective data revealed that among these individuals, 0.7% were HBsAg carriers and 7.7% had anti-HBc. This results in an 8-fold discrepancy between anti-HBc seroprevalence (8.7%) and awareness of past infection (1.1%), indicating that HBV remains largely undiagnosed in Serbia, pre-dominantly asymptomatic. The exceptionally low infectious dose of HBV [as low as 3.5 IU/ml (66)] further heightens the risk of hidden transmission and delayed diagnosis until severe complications (cirrhosis, hepatocellular carcinoma). The sharp increase in anti-HBc seroprevalence after age 50 provides a clear epidemiological rationale for one-time HBsAg and anti-HBc testing for all individuals aged ≥40 years upon any contact with primary healthcare. Undiagnosed chronic infections in older age groups, particularly among NINV and NIV individuals, represent a persistent reservoir for transmission and a future burden of progressive liver disease.

The two individuals in the “infected and vaccinated” (IV) category were seronegative for all markers. While this may reflect inaccurate self-reporting, it could also represent a state of “serologically silent” past infection with waned antibody levels, a phenomenon carrying a known risk of reactivation (67–70).

The relatively high frequency of anti-HBs among NINV children is of particular interest. With the small sample size, this indicator could reflect several phenomena: the possibility of asymptomatic infection leading to subsequent immunity formation; passive transfer of Abs from mother to child; or the presence of unrecorded vaccination not reflected in the records. This result highlights the difficulty of accurately classifying immune status based solely on survey data.

### 4.6 Comparison with other national studies: confirmation of trends and identification of systemic issues

A large-scale study in Vojvodina (13) revealed a similar age-related dynamic: 80.7% anti-HBs seroprevalence in individuals aged 1–19 years vs. 17.7% in adults aged 20–59 years, mirroring the sharp decline after age 29 in our data. This confirms that the generational immune gap is a systemic phenomenon across Serbia, and not a feature of a specific sample.

The data from Vojvodina (13) further support our conclusions: 99.8% of anti-HBs-positive young people lacked anti-HBc, independently confirming that high immunity in youth is vaccine-induced, not from natural infection. Conversely, 65.9% of older adults individuals in Vojvodina had a resolved infection profile (anti-HBc+/anti-HBs+), aligning with our observation of peak anti-HBc in the 70+ group (22.2%) and supporting a persistent reservoir of historical infection.

Anti-HBc is a classical marker present in all phases of HBV infection, including ~90% of occult cases where HBsAg may be absent (43, 44, 71, 72). The dynamic of HBcAg expression and its secretion in the serum is still not completely understood. Simultaneous increases in anti-HBc with alanine aminotransferase and aspartate aminotransferase have been reported (73, 74).

The “anti-HBc only” profile in 3.9% of our population may indicate resolved infection with waning anti-HBs, occult HBV, or other region-specific explanations (75, 76); such individuals (HBsAg-negative, anti-HBc-positive) require monitoring (35).

The comparison also reveals common systemic problems. The alarmingly low level of confirmed immunity among the vaccinated identified in our study (anti-HBs detected in only 49.9%) mirrors data from a study of healthcare workers in the city of Šabac (77). Despite 100% vaccination coverage and 89.5% completion of the full course, 15% of medical workers had Ab levels below the protective threshold. This indicates that the problem of a weak immune response and lack of seroconversion after vaccination is not random but widespread, creating vulnerable groups among formally protected individuals. This observation reinforces our recommendation for the implementation of mandatory post-vaccination serological control, primarily in occupational risk groups.

In Serbia, routine HBV screening is only performed in pregnant women (9th month of pregnancy) (78). The 8-fold gap between seroprevalence and awareness likely reflects a lack of routine screening or low awareness among primary care physicians (79, 80).

The polarized distribution of anti-HBs levels (most individuals either lacking protective antibodies or having very high levels) suggests variable vaccine efficacy, influenced by age, comorbidities, and other factors (obesity, smoking, male sex, immunosuppression, chronic liver/kidney disease, diabetes) (35, 60, 81, 82). We are not aware of other national or global studies discussing this polarized seropositivity pattern.

Comparison with representative studies from other Serbian regions allows us to assert that our conclusions reflect stable nationwide patterns: successful infection control among younger generations thanks to vaccination; the persistent burden of historical infection among the older adults; and the presence of significant gaps in protection among the adult population, exacerbated by the problem of insufficient post-vaccination immune response. This confirms the necessity for targeted actions at the national level aimed at screening older age groups and optimizing the adult immunization strategy.

### 4.7 Limitations of the study

This study has several limitations that should be considered when interpreting its results. First, concerning information bias, key variables (including vaccination status and history of hepatitis B infection) were primarily collected via self-reporting. This approach is inherently susceptible to recall bias, particularly among older adults who may not accurately remember vaccinations or past subclinical infections. The inability to systematically verify self-reported data with medical records for most participants likely led to an underestimation of true vaccination coverage and an overestimation of the seroprevalence of natural infection, as some vaccinated individuals might have reported as unvaccinated, and vice versa. This bias may have affected the accuracy of age-stratified analyses, especially in cohorts born before the era of universal vaccination.

Second, the study did not collect detailed data on specific behavioral risk factors beyond a history of surgery or blood transfusion. Information on other known risk factors for HBV infection (intravenous drug use, high-risk sexual behavior, travel history to endemic regions, household contact with chronic carriers) was not available. The absence of these data limits our ability to perform a more nuanced risk-factor analysis and may have resulted in unmeasured confounding when interpreting the observed seroprevalence patterns.

Finally, sample representativeness and size present notable constraints. The voluntary nature of participation, conducted via a web application, likely introduced a selection bias, as evidenced by the overrepresentation of female and middle-aged participants. Importantly, the study used a randomized quota sampling design from self-selected volunteers rather than a simple random sample of the general population. This may limit the generalizability of absolute prevalence estimates to the entire population of Belgrade. Furthermore, the small sample sizes in pediatric groups increase the uncertainty around seroprevalence estimates in these critical age cohorts. Therefore, conclusions regarding herd immunity in these younger age groups, which are central to vaccination policy, should be drawn with particular caution. Despite these limitations, this study provides valuable, population-based serological data that reflect the complex interplay of natural infection and vaccination over several decades in Belgrade.

## 5 Conclusions

Universal childhood hepatitis B vaccination in Serbia has been associated with a marked reduction in HBV transmission, as reflected by low HBsAg prevalence (1.0%) and an anti-HBc seroprevalence of 8.7%, which classifies Serbia as a low-endemicity country. A clear age-dependent pattern indicates successful protection of the younger generation, although the small sample size in pediatric subgroups precludes precise estimation of infection markers in children and adolescents.

Three major gaps persist. First, there is an 8-fold discrepancy between serological evidence of past infection (anti-HBc 8.7%) and population awareness (1.1%), indicating a large burden of undiagnosed chronic infections, particularly among adults aged ≥40 years. Second, only 50.4% of vaccinated individuals have detectable protective anti-HBs, highlighting a clinically significant non-response problem. Third, adults aged ≥30 years, especially retirees and education workers, remain largely susceptible, with vaccination coverage below 10% in those ≥60 years.

To achieve HBV elimination, Serbia should implement age-targeted screening for adults ≥40 years to identify undiagnosed chronic infections. For adult vaccination programmes (e.g., for education workers), we recommend pre-vaccination testing (HBsAg, anti-HBc, anti-HBs) to avoid vaccinating those already immune or chronically infected, followed by post-vaccination serology (anti-HBs) to confirm protective response. Additionally, the cost-effectiveness of an adolescent booster dose should be evaluated. Larger pediatric studies are needed to accurately assess HBV marker prevalence in children. Implementing these measures alongside existing childhood immunization programmes would consolidate progress and advance hepatitis B elimination in Serbia.

## Data Availability

The datasets presented in this article are not readily available because don't have restrictions. Requests to access the datasets should be directed to tvildorm@gmail.com.
