# Supplementary material for: Population immunity to hepatitis B virus and infection marker seroprevalence in Belgrade, Serbia
Source: Front Public Health. 2026 Jun 17;14:1819814. doi: 10.3389/fpubh.2026.1819814 (PMC13319082; doi:10.3389/fpubh.2026.1819814)
Supplement: Supplementary file 1 [file Data_Sheet_1.docx]

**Supplementary Table S1.** Hepatitis B viral markers by field of activity.

| **Field of Activity** | **N** | **HBsAg** | | | **Anti-HBc** | | | **Anti-HBs** | | |
| --- | --- | --- | --- | --- | --- | --- | --- | --- | --- | --- |
|  |  | **n** | **%** | **95% CI** | **n** | **%** | **95% CI** | **n** | **%** | **95% CI** |
| Medicine | 541 | 3 | 0.6 | 0.2 - 1.6 | 35 | 6.5 | 4.7 - 8.9 | 243 | 44.9* | 40.8 - 49.1 |
| Science | 138 | 1 | 0.7 | 0.1 - 4.0 | 9 | 6.5 | 3.5 - 11.9 | 31 | 22.5 | 16.3 - 30.1 |
| Business | 198 | 1 | 0.5 | 0.1 - 2.8 | 13 | 6.6 | 3.9 - 10.9 | 16 | 8.1^#^ | 5.0 - 12.7 |
| Education | 138 | 0 | 0.0 | 0.0 - 2.6 | 11 | 8.0 | 4.5 - 13.7 | 16 | 11.6^#^ | 7.3 - 18.0 |
| Art/creativity | 68 | 0 | 0.0 | 0.0 - 5.3 | 5 | 7.4 | 3.2 - 16.1 | 11 | 16.2 | 9.3 - 26.7 |
| Production | 47 | 1 | 2.1 | 0.4 - 11.1 | 2 | 4.3 | 1.2 - 14.2 | 6 | 12.8 | 6.0 - 25.2 |
| Transport | 36 | 1 | 2.8 | 0.5 - 14.2 | 4 | 11.1 | 4.4 - 25.3 | 3 | 8.3 | 2.9 - 21.8 |
| Military service | 19 | 0 | 0.0 | 0.0 - 17.6 | 4 | 21.1 | 8.5 - 43.3 | 5 | 26.3 | 11.8 - 48.8 |
| Public service | 190 | 3 | 1.6 | 0.5 - 4.5 | 17 | 8.9 | 5.7 - 13.9 | 18 | 9.5^#^ | 6.1 - 14.5 |
| Office | 152 | 3 | 2.0 | 0.7 - 5.6 | 12 | 7.9 | 4.6 - 13.3 | 11 | 7.2^#^ | 4.1 - 12.5 |
| Unemployed | 112 | 3 | 2.7 | 0.9 - 7.6 | 9 | 8.0 | 4.3 - 14.6 | 18 | 16.1 | 10.4 - 24.0 |
| Preschooler | 16 | 0 | 0.0 | 0.0 - 20.6 | 1 | 6.3 | 1.1 - 28.3 | 10 | 62.5* | 38.6 - 81.5 |
| Miscellaneous | 179 | 1 | 0.6 | 0.1 - 3.1 | 14 | 7.8 | 4.7 - 12.7 | 15 | 8.4^#^ | 5.1 - 13.4 |
| Schoolchild | 91 | 0 | 0.0 | 0.0 - 4.0 | 8 | 8.8 | 4.5 - 16.4 | 26 | 28.6 | 20.3 - 38.6 |
| Student | 85 | 0 | 0.0 | 0.0 - 4.2 | 3 | 3.5 | 1.2 - 9.9 | 42 | 49.4* | 39.0 - 59.8 |
| Retiree | 363 | 7 | 1.9 | 0.9 - 3.9 | 66 | 18.2* | 14.6 - 22.5 | 57 | 15.7^#^ | 12.3 - 19.8 |
| Tourism | 17 | 0 | 0.0 | 0.0 - 19.5 | 0 | 0.0 | 0.0 - 19.5 | 2 | 11.8 | 3.3 - 34.3 |
| Agriculture | 4 | 0 | 0.0 | 0.0 - 60.2 | 0 | 0.0 | 0.0 - 60.2 | 0 | 0.0 | 0.0 - 60.2 |
| IT | 139 | 1 | 0.7 | 0.1 - 4.0 | 7 | 5.0 | 2.5 - 10.0 | 17 | 12.2^#^ | 7.8 - 18.7 |
| Total | 2533 | 25 | 1.0 | 0.7 - 1.5 | 220 | 8.7 | 7.7 - 9.8 | 547 | 21.6 | 20.0 - 23.2 |

Note: * significantly higher than the total value; ^#^ significantly lower than the total value; p<0.05 for all comparisons.
