# Supplementary material for: Population immunity to hepatitis B virus and infection marker seroprevalence in Belgrade, Serbia
Source: Front Public Health. 2026 Jun 17;14:1819814. doi: 10.3389/fpubh.2026.1819814 (PMC13319082; doi:10.3389/fpubh.2026.1819814)
Supplement: Supplementary file 2 [file Data_Sheet_2.docx]

**Supplementary Table S2.** Hepatitis B markers in volunteers by intervention history (surgical, blood transfusion).

| **History of Surgery or Blood Transfusion** | **N** | **HBsAg** | | | **Anti-HBc** | | | **Anti-HBs** | | |
| --- | --- | --- | --- | --- | --- | --- | --- | --- | --- | --- |
|  |  | **n** | **%** | **95% CI** | **n** | **%** | **95% CI** | **n** | **%** | **95% CI** |
| Yes | 1185 | 10 | 0.8 | 0.5 - 1.5 | 116 | 9.8 | 8.2 - 11.6 | 254 | 21.4 | 19.2 – 23.9 |
| No | 1246 | 15 | 1.2 | 0.7 - 2.0 | 100 | 8.0 | 6.6 - 9.7 | 270 | 21.7 | 19.5 – 24.0 |
