# Supplementary material for: Population immunity to hepatitis B virus and infection marker seroprevalence in Belgrade, Serbia
Source: Front Public Health. 2026 Jun 17;14:1819814. doi: 10.3389/fpubh.2026.1819814 (PMC13319082; doi:10.3389/fpubh.2026.1819814)
Supplement: Supplementary file 3 [file Data_Sheet_3.docx]

**Supplementary Table S3.** Hepatitis B self-report history by volunteer age group.

| **Age Group, years** | **N** | **Experienced hepatitis B** | | | **Never Experienced hepatitis B** | | | **Information not available** | | |
| --- | --- | --- | --- | --- | --- | --- | --- | --- | --- | --- |
|  |  | **n** | **%** | **95% CI** | **n** | **%** | **95% CI** | **n** | **%** | **95% CI** |
| 1 - 17 | 118 | 0 | 0.0 | 0.0 - 3.1 | 118 | 100 | 96.8 - 100.0 | 0 | 0.0 | 0.0 - 3.1 |
| 1-5 | 13 | 0 | 0.0 | 0.0 - 24.7 | 13 | 100 | 77.2 - 100.0 | 0 | 0.0 | 0.0 - 24.7 |
| 6-11 | 43 | 0 | 0.0 | 0.0 - 8.2 | 43 | 100 | 91.8 - 100.0 | 0 | 0.0 | 0.0 - 8.2 |
| 12-17 | 62 | 0 | 0.0 | 0.0 - 5.8 | 62 | 100 | 94.2 - 100.0 | 0 | 0.0 | 0.0 - 5.8 |
| 18-29 | 249 | 0 | 0.0 | 0.0 - 1.5 | 248 | 99.6 | 97.8 - 99.9 | 1 | 0.4 | 0.1 - 2.2 |
| 30-39 | 501 | 2 | 0.4 | 0.1 - 1.4 | 493 | 98.4 | 96.9 - 99.2 | 6 | 1.2 | 0.5 - 2.6 |
| 40-49 | 688 | 2 | 0.3 | 0.1 - 1.1 | 675 | 98.1 | 96.8 - 98.9 | 11 | 1.6 | 0.9 - 2.8 |
| 50-59 | 468 | 7 | 1.5 | 0.7 - 3.1 | 457 | 97.6 | 95.8 - 98.7 | 4 | 0.9 | 0.3 - 2.2 |
| 60-69 | 320 | 6 | 1.9 | 0.9 - 4.0 | 304 | 95.0 | 92.0 - 96.9 | 10 | 3.1 | 1.7 - 5.7 |
| 70+ | 189 | 11 | 5.8* | 3.3 - 10.1 | 173 | 91.5^#^ | 86.7 - 94.7 | 5 | 2.6 | 1.1 - 6.0 |
| Total | 2533 | 28 | 1.1 | 0.8 - 1.6 | 2468 | 97.4 | 96.7 - 98.0 | 37 | 1.5 | 1.1 - 2.0 |

Note: * significantly higher than the total value; ^#^ significantly lower than the total value; p<0.05 for all comparisons.
