# Supplementary material for: Population immunity to hepatitis B virus and infection marker seroprevalence in Belgrade, Serbia
Source: Front Public Health. 2026 Jun 17;14:1819814. doi: 10.3389/fpubh.2026.1819814 (PMC13319082; doi:10.3389/fpubh.2026.1819814)
Supplement: Supplementary file 4 [file Data_Sheet_4.docx]

**Supplementary Table S4.** Vaccination coverage (hepatitis B) by volunteer activity.

| **Field of Activity** | **N** | **Vaccinated against HBV** | | |
| --- | --- | --- | --- | --- |
|  |  | **n** | **%** | **95% CI** |
| Medicine | 517 | 379 | 73.3* | 69.3 - 76.9 |
| Education | 124 | 20 | 16.1^#^ | 10.7 - 23.6 |
| Preschooler | 12 | 10 | 83.3* | 55.2 - 95.3 |
| Schoolchild | 90 | 74 | 82.2* | 73.1 - 88.8 |
| Students | 82 | 46 | 56.1* | 45.3 - 66.3 |
| Retirees | 345 | 16 | 4.6^#^ | 2.9 - 7.4 |
| Others | 1137 | 166 | 14.6^#^ | 12.7 - 16.8 |
| Total | 2307 | 711 | 30.8 | 29.0 - 32.7 |

Note: * significantly higher than the total value; ^#^ significantly lower than the total value; p<0.05 for all comparisons. As before, only those volunteers who were certain about the presence or absence of hepatitis B vaccination were considered.
