# Supplementary material for: Population immunity to hepatitis B virus and infection marker seroprevalence in Belgrade, Serbia
Source: Front Public Health. 2026 Jun 17;14:1819814. doi: 10.3389/fpubh.2026.1819814 (PMC13319082; doi:10.3389/fpubh.2026.1819814)
Supplement: Supplementary file 5 [file Data_Sheet_5.docx]

**Supplementary Table S5.** Volunteer history (infection, vaccination) by age group.

| Age Group, years | N | INV | | | IV | | | NINV | | | NIV | | |
| --- | --- | --- | --- | --- | --- | --- | --- | --- | --- | --- | --- | --- | --- |
|  |  | n | % | 95% CI | n | % | 95% CI | n | % | 95% CI | n | % | 95% CI |
| 1 - 17 | 113 | 0 | 0.0 | 0.0 - 3.2 | 0 | 0.0 | 0.0 - 3.2 | 20 | 17.7^#^ | 11.8 - 25.8 | 93 | 82.3* | 74.2 - 88.2 |
| 1-5 | 11 | 0 | 0.0 | 0.0 - 28.5 | 0 | 0.0 | 0.0 - 28.5 | 2 | 18.2^#^ | 5.1 - 47.7 | 9 | 81.8* | 52.3 - 94.9 |
| 6-11 | 40 | 0 | 0.0 | 0.0 - 8.8 | 0 | 0.0 | 0.0 - 8.8 | 10 | 25.0^#^ | 14.2 - 40.2 | 30 | 75.0* | 59.8 - 85.8 |
| 12-17 | 62 | 0 | 0.0 | 0.0 - 5.8 | 0 | 0.0 | 0.0 - 5.8 | 8 | 12.9^#^ | 6.7 - 23.4 | 54 | 87.1* | 76.6 - 93.3 |
| 18-29 | 222 | 0 | 0.0 | 0.0 - 1.6 | 0 | 0.0 | 0.0 - 1.6 | 75 | 33.8^#^ | 27.9 - 40.2 | 147 | 66.2* | 59.8 - 72.1 |
| 30-39 | 436 | 2 | 0.5 | 0.1 - 1.7 | 0 | 0.0 | 0.0 - 0.8 | 275 | 63.1 | 58.4 - 67.5 | 159 | 36.5 | 32.1 - 41.1 |
| 40-49 | 617 | 2 | 0.3 | 0.1 - 1.2 | 0 | 0.0 | 0.0 - 0.6 | 447 | 72.4 | 68.8 - 75.8 | 168 | 27.2 | 23.9 - 30.9 |
| 50-59 | 428 | 7 | 1.6 | 0.8 - 3.3 | 0 | 0.0 | 0.0 - 0.9 | 313 | 73.1 | 68.7 - 77.1 | 108 | 25.2 | 21.4 - 29.6 |
| 60-69 | 293 | 5 | 1.7 | 0.7 - 3.9 | 1 | 0.3 | 0.1 - 1.9 | 262 | 89.4* | 85.4 - 92.4 | 25 | 8.5^#^ | 5.8 - 12.3 |
| 70+ | 173 | 10 | 5.8* | 3.2 - 10.3 | 1 | 0.6 | 0.1 - 3.2 | 154 | 89.0* | 83.5 - 92.9 | 8 | 4.6^#^ | 2.4 - 8.9 |
| Total | 2282 | 26 | 1.1 | 0.8 - 1.7 | 2 | 0.1 | 0.0 - 0.3 | 1546 | 67.7 | 65.8 - 69.6 | 708 | 31.0 | 29.2 - 33.0 |

Note: * significantly higher than the total value; ^#^ significantly lower than the total value; p<0.05 for all comparisons.
