# Supplementary material for: Population immunity to hepatitis B virus and infection marker seroprevalence in Belgrade, Serbia
Source: Front Public Health. 2026 Jun 17;14:1819814. doi: 10.3389/fpubh.2026.1819814 (PMC13319082; doi:10.3389/fpubh.2026.1819814)
Supplement: Supplementary file 6 [file Data_Sheet_6.docx]

**Supplementary Table S6.** HBsAg detection frequency by age group and volunteer history (infection, vaccination).

| Age Group, years | INV | | | | IV | | | | NINV | | | | NIV | | | |
| --- | --- | --- | --- | --- | --- | --- | --- | --- | --- | --- | --- | --- | --- | --- | --- | --- |
|  | N | n | % | 95% CI | N | n | % | 95% CI | N | n | % | 95% CI | N | n | % | 95% CI |
| 1 - 17 | 0 | 0 | 0.0 | 0.0 - 0.0 | 0 | 0 | 0.0 | 0.0 - 0.0 | 20 | 0 | 0.0 | 0.0 - 16.8 | 93 | 0 | 0.0 | 0.0 - 3.9 |
| 1-5 | 0 | 0 | 0.0 | 0.0 - 0.0 | 0 | 0 | 0.0 | 0.0 - 0.0 | 2 | 0 | 0.0 | 0.0 - 84.2 | 9 | 0 | 0.0 | 0.0 - 33.6 |
| 6-11 | 0 | 0 | 0.0 | 0.0 - 0.0 | 0 | 0 | 0.0 | 0.0 - 0.0 | 10 | 0 | 0.0 | 0.0 - 30.8 | 30 | 0 | 0.0 | 0.0 - 11.6 |
| 13-17 | 0 | 0 | 0.0 | 0.0 - 0.0 | 0 | 0 | 0.0 | 0.0 - 0.0 | 8 | 0 | 0.0 | 0.0 - 36.9 | 54 | 0 | 0.0 | 0.0 - 6.6 |
| 18-29 | 0 | 0 | 0.0 | 0.0 - 0.0 | 0 | 0 | 0.0 | 0.0 - 0.0 | 75 | 0 | 0.0 | 0.0 - 4.8 | 147 | 0 | 0.0 | 0.0 - 2.5 |
| 30-39 | 2 | 1 | 50.0 | 9.5 - 90.5 | 0 | 0 | 0.0 | 0.0 - 0.0 | 275 | 1 | 0.4 | 0.1 - 2.0 | 159 | 0 | 0.0 | 0.0 - 2.3 |
| 40-49 | 2 | 0 | 0.0 | 0.0 - 84.2 | 0 | 0 | 0.0 | 0.0 - 0.0 | 447 | 1 | 0.2 | 0.0 - 1.3 | 168 | 3 | 1.8 | 0.6 - 5.1 |
| 50-59 | 7 | 3 | 42.9 | 15.8 - 75.0 | 0 | 0 | 0.0 | 0.0 - 0.0 | 313 | 2 | 0.6 | 0.2 - 2.3 | 108 | 2 | 1.9 | 0.5 - 6.5 |
| 60-69 | 5 | 2 | 40.0 | 11.8 - 76.9 | 1 | 0 | 0.0 | 0.0 - 97.5 | 262 | 5 | 1.9 | 0.8 - 4.4 | 25 | 0 | 0.0 | 0.0 - 13.7 |
| 70+ | 10 | 0 | 0.0 | 0.0 - 30.8 | 1 | 0 | 0.0 | 0.0 - 97.5 | 154 | 2 | 1.3 | 0.4 - 4.6 | 8 | 0 | 0.0 | 0.0 - 36.9 |
| Total | 26 | 6 | 23.1 | 11.0 - 42.1 | 2 | 0 | 0.0 | 0.0 - 84.2 | 1546 | 11 | 0.7 | 0.4 - 1.3 | 708 | 5 | 0.7 | 0.3 - 1.6 |
