# Supplementary material for: Population immunity to hepatitis B virus and infection marker seroprevalence in Belgrade, Serbia
Source: Front Public Health. 2026 Jun 17;14:1819814. doi: 10.3389/fpubh.2026.1819814 (PMC13319082; doi:10.3389/fpubh.2026.1819814)
Supplement: Supplementary file 7 [file Data_Sheet_7.docx]

**Supplementary Table S7.** Anti-HBc detection frequency by age group and volunteer history (infection, vaccination).

| **Age Group, years** | **INV** | | | | **IV** | | | | **NINV** | | | | **NIV** | | | |
| --- | --- | --- | --- | --- | --- | --- | --- | --- | --- | --- | --- | --- | --- | --- | --- | --- |
|  | **N** | **n** | **%** | **95% CI** | **N** | **n** | **%** | **95% CI** | **N** | **n** | **%** | **95% CI** | **N** | **n** | **%** | **95% CI** |
| 1 - 17 | 0 | 0 | 0.0 | 0.0 - 0.0 | 0 | 0 | 0.0 | 0.0 - 0.0 | 20 | 3 | 15.0 | 5.2 - 36.0 | 93 | 6 | 6.5 | 3.0 - 13.4 |
| 1-5 | 0 | 0 | 0.0 | 0.0 - 0.0 | 0 | 0 | 0.0 | 0.0 - 0.0 | 2 | 0 | 0.0 | 0.0 - 84.2 | 9 | 1 | 11.1 | 2.0 - 43.5 |
| 6-11 | 0 | 0 | 0.0 | 0.0 - 0.0 | 0 | 0 | 0.0 | 0.0 - 0.0 | 10 | 2 | 20.0 | 5.7 - 51.0 | 30 | 2 | 6.7 | 1.8 - 21.3 |
| 13-17 | 0 | 0 | 0.0 | 0.0 - 0.0 | 0 | 0 | 0.0 | 0.0 - 0.0 | 8 | 1 | 12.5 | 2.2 - 47.1 | 54 | 3 | 5.6 | 1.9 - 15.1 |
| 18-29 | 0 | 0 | 0.0 | 0.0 - 0.0 | 0 | 0 | 0.0 | 0.0 - 0.0 | 75 | 3 | 4.0 | 1.4 - 11.1 | 147 | 6 | 4.1 | 1.9 - 8.6 |
| 30-39 | 2 | 2 | 100 | 34.2 - 100.0 | 0 | 0 | 0.0 | 0.0 - 0.0 | 275 | 12 | 4.4 | 2.5 - 7.5 | 159 | 11 | 6.9 | 3.9 - 12.0 |
| 40-49 | 2 | 2 | 100 | 34.2 - 100.0 | 0 | 0 | 0.0 | 0.0 - 0.0 | 447 | 28 | 6.3 | 4.4 - 8.9 | 168 | 7 | 4.2 | 2.0 - 8.3 |
| 50-59 | 7 | 7 | 100 | 64.6 - 100.0 | 0 | 0 | 0.0 | 0.0 - 0.0 | 313 | 26 | 8.3 | 5.7 - 11.9 | 108 | 10 | 9.3 | 5.1 - 16.2 |
| 60-69 | 5 | 4 | 80.0 | 37.6 - 96.4 | 1 | 0 | 0.0 | 0.0 - 97.5 | 262 | 31 | 11.8 | 8.5 - 16.3 | 25 | 3 | 12.0 | 4.2 - 30.0 |
| 70+ | 10 | 7 | 70.0 | 39.7 - 89.2 | 1 | 0 | 0.0 | 0.0 - 97.5 | 154 | 33 | 21.4* | 15.7 - 28.6 | 8 | 0 | 0.0 | 0.0 - 36.9 |
| Total | 26 | 22 | 84.6 | 66.5 - 93.9 | 2 | 0 | 0.0 | 0.0 - 84.2 | 1546 | 136 | 8.8 | 7.5 - 10.3 | 708 | 43 | 6.1 | 4.5 - 8.1 |

Note: * significantly higher than the total value; ^#^ significantly lower than the total value; p<0.05 for all comparisons.
