# Supplementary material for: Population immunity to hepatitis B virus and infection marker seroprevalence in Belgrade, Serbia
Source: Front Public Health. 2026 Jun 17;14:1819814. doi: 10.3389/fpubh.2026.1819814 (PMC13319082; doi:10.3389/fpubh.2026.1819814)
Supplement: Supplementary file 8 [file Data_Sheet_8.docx]

**Supplementary Table S8.** Anti-HBs detection frequency by age group and volunteer history (infection, vaccination).

| **Age Group, years** | **INV** | | | | **IV** | | | | **NINV** | | | | **NIV** | | | |
| --- | --- | --- | --- | --- | --- | --- | --- | --- | --- | --- | --- | --- | --- | --- | --- | --- |
|  | **N** | **n** | **%** | **95% CI** | **N** | **n** | **%** | **95% CI** | **N** | **n** | **%** | **95% CI** | **N** | **n** | **%** | **95% CI** |
| 1 - 17 | 0 | 0 | 0.0 | 0.0 - 0.0 | 0 | 0 | 0.0 | 0.0 - 0.0 | 20 | 5 | 25.0* | 11.2 - 46.9 | 93 | 33 | 35.5^#^ | 26.5 - 45.6 |
| 1-5 | 0 | 0 | 0.0 | 0.0 - 0.0 | 0 | 0 | 0.0 | 0.0 - 0.0 | 2 | 2 | 100 | 34.2 - 100.0 | 9 | 6 | 66.7 | 35.4 - 87.9 |
| 6-11 | 0 | 0 | 0.0 | 0.0 - 0.0 | 0 | 0 | 0.0 | 0.0 - 0.0 | 10 | 1 | 10.0 | 1.8 - 40.4 | 30 | 14 | 46.7 | 30.2 - 63.9 |
| 13-17 | 0 | 0 | 0.0 | 0.0 - 0.0 | 0 | 0 | 0.0 | 0.0 - 0.0 | 8 | 2 | 25.0 | 7.1 - 59.1 | 54 | 13 | 24.1^#^ | 14.6 - 36.9 |
| 18-29 | 0 | 0 | 0.0 | 0.0 - 0.0 | 0 | 0 | 0.0 | 0.0 - 0.0 | 75 | 39 | 52.0* | 40.9 - 62.9 | 147 | 86 | 58.5 | 50.4 - 66.2 |
| 30-39 | 2 | 1 | 50.0 | 9.5 - 90.5 | 0 | 0 | 0.0 | 0.0 - 0.0 | 275 | 8 | 2.9^#^ | 1.5 - 5.6 | 159 | 86 | 54.1 | 46.3 - 61.6 |
| 40-49 | 2 | 2 | 100 | 34.2 - 100.0 | 0 | 0 | 0.0 | 0.0 - 0.0 | 447 | 15 | 3.4^#^ | 2.0 - 5.5 | 168 | 83 | 49.4 | 41.9 - 56.9 |
| 50-59 | 7 | 2 | 28.6 | 8.2 - 64.1 | 0 | 0 | 0.0 | 0.0 - 0.0 | 313 | 18 | 5.8 | 3.7 - 8.9 | 108 | 52 | 48.1 | 39.0 - 57.5 |
| 60-69 | 5 | 2 | 40.0 | 11.8 - 76.9 | 1 | 0 | 0.0 | 0.0 - 97.5 | 262 | 22 | 8.4 | 5.6 - 12.4 | 25 | 13 | 52.0 | 33.5 - 70.0 |
| 70+ | 10 | 5 | 50.0 | 23.7 - 76.3 | 1 | 0 | 0.0 | 0.0 - 97.5 | 154 | 26 | 16.9* | 11.8 - 23.6 | 8 | 4 | 50.0 | 21.5 - 78.5 |
| Total | 26 | 12 | 46.2 | 28.8 - 64.5 | 2 | 0 | 0.0 | 0.0 - 84.2 | 1546 | 133 | 8.6 | 7.3 - 10.1 | 708 | 357 | 50.4 | 46.7 - 54.1 |

Note: * significantly higher than the total value; ^#^ significantly lower than the total value; p<0.05 for all comparisons.
