# Supplementary material for: Population immunity to hepatitis B virus and infection marker seroprevalence in Belgrade, Serbia
Source: Front Public Health. 2026 Jun 17;14:1819814. doi: 10.3389/fpubh.2026.1819814 (PMC13319082; doi:10.3389/fpubh.2026.1819814)
Supplement: Supplementary file 9 [file Data_Sheet_9.docx]

**Supplementary Table S9.** Anti-HBs levels by volunteer age group.

| **Age Group, years** | **N** | **0-9  (mIU/ml)** | | | **10-50  (mIU/ml)** | | | **51-100  (mIU/ml)** | | | **101-149  (mIU/ml)** | | | **≥ 150 (mIU/ml)** | | |
| --- | --- | --- | --- | --- | --- | --- | --- | --- | --- | --- | --- | --- | --- | --- | --- | --- |
|  |  | **n** | **%** | **95% CI** | **n** | **%** | **95% CI** | **n** | **%** | **95% CI** | **n** | **%** | **95% CI** | **n** | **%** | **95% CI** |
| 1 - 17 | 118 | 76 | 64.4^#^ | 55.4 - 72.5 | 24 | 20.3* | 14.1 - 28.5 | 8 | 6.8 | 3.5 - 12.8 | 6 | 5.1 | 2.4 - 10.7 | 4 | 3.4 | 1.3 - 8.4 |
| 1-5 | 13 | 3 | 23.1^#^ | 8.2 - 50.3 | 6 | 46.2* | 23.2 - 70.9 | 1 | 7.7 | 1.4 - 33.3 | 2 | 15.4* | 4.3 - 42.2 | 1 | 7.7 | 1.4 - 33.3 |
| 6-11 | 43 | 26 | 60.5^#^ | 45.6 - 73.6 | 10 | 23.3* | 13.2 - 37.7 | 3 | 7.0 | 2.4 - 18.6 | 1 | 2.3 | 0.4 - 12.1 | 3 | 7.0 | 2.4 - 18.6 |
| 12-17 | 62 | 47 | 75.8 | 63.8 - 84.8 | 8 | 12.9 | 6.7 - 23.4 | 4 | 6.5 | 2.5 - 15.4 | 3 | 4.8 | 1.7 - 13.3 | 0 | 0.0 | 0.0 - 5.8 |
| 18-29 | 249 | 104 | 41.8^#^ | 35.8 - 48.0 | 54 | 21.7* | 17.0 - 27.2 | 25 | 10.0 | 6.9 - 14.4 | 9 | 3.6 | 1.9 - 6.7 | 57 | 23.0* | 18.1 - 28.5 |
| 30-39 | 501 | 399 | 79.6 | 75.9 - 82.9 | 36 | 7.2 | 5.2 - 9.8 | 17 | 3.4 | 2.1 - 5.4 | 9 | 1.8 | 0.9 - 3.4 | 40 | 8.0 | 5.9 - 10.7 |
| 40-49 | 688 | 585 | 85.0* | 82.2 - 87.5 | 46 | 6.7 | 5.0 - 8.8 | 15 | 2.2 | 1.3 - 3.6 | 6 | 0.9 | 0.4 - 1.9 | 36 | 5.2 | 3.8 - 7.2 |
| 50-59 | 468 | 392 | 83.8 | 80.1 - 86.8 | 33 | 7.1 | 5.1 - 9.7 | 9 | 1.9 | 1.0 - 3.6 | 3 | 0.6 | 0.2 - 1.9 | 31 | 6.6 | 4.7 - 9.2 |
| 60-69 | 320 | 278 | 86.9* | 82.7 - 90.1 | 17 | 5.3 | 3.3 - 8.3 | 5 | 1.6 | 0.7 - 3.6 | 2 | 0.6 | 0.2 - 2.2 | 18 | 5.6 | 3.6 - 8.7 |
| 70+ | 189 | 152 | 80.4 | 74.2 - 85.5 | 12 | 6.3 | 3.7 - 10.8 | 10 | 5.3 | 2.9 - 9.5 | 5 | 2.6 | 1.1 - 6.0 | 10 | 5.3 | 2.9 - 9.5 |
| Total | 2533 | 1986 | 78.4 | 76.8 - 80.0 | 222 | 8.8 | 7.7 - 9.9 | 89 | 3.5 | 2.9 - 4.3 | 40 | 1.6 | 1.2 - 2.1 | 196 | 7.7 | 6.8 - 8.8 |

Note: * significantly higher than the total value; ^#^ significantly lower than the total value; p<0.05 for all comparisons.
